# Effect of High Temperature Stress During the Reproductive Stage on Grain Yield and Nutritional Quality of Lentil (*Lens culinaris* Medikus)

**DOI:** 10.3389/fnut.2022.857469

**Published:** 2022-04-15

**Authors:** Hasnae Choukri, Noureddine El Haddad, Khawla Aloui, Kamal Hejjaoui, Adil El-Baouchi, Abdelaziz Smouni, Dil Thavarajah, Fouad Maalouf, Shiv Kumar

**Affiliations:** ^1^Laboratoire de Biotechnologie et de Physiologie Végétales, Faculté des Sciences, Centre de Recherche BioBio, University Mohammed V, Rabat, Morocco; ^2^International Center for Agricultural Research in the Dry Areas, Rabat, Morocco; ^3^Laboratory of Ecology and Environment, Ben M'Ski Faculty of Sciences, University Hassan II, Casablanca, Morocco; ^4^African Integrated Plant and Soil Research Group, AgroBioSciences, University Mohammed VI Polytechnic, Ben Guerir, Morocco; ^5^Plant and Environmental Sciences, 113 Biosystems Research Complex, Clemson University, Clemson, SC, United States; ^6^International Center for Agricultural Research in the Dry Areas, Beirut, Lebanon

**Keywords:** lentil, biofortification, heat, grain yield, crude protein, iron and zinc, phytic acid, cooking time

## Abstract

High temperature during the reproductive stage limits the growth and development of lentil (*Lens culinaris* Medikus). The reproductive and seed filling periods are the most sensitive to heat stress, resulting in limited yield and nutritional quality. Climate change causes frequent incidents of heat stress for global food crop production. This study aimed to assess the impact of high temperature during the reproductive stage of lentil on grain yield, nutritional value, and cooking quality. Thirty-six lentil genotypes were evaluated under controlled conditions for their high temperature response. Genotypic variation was significant (*p* < 0.001) for all the traits under study. High temperature-induced conditions reduced protein, iron (Fe) and zinc (Zn) concentrations in lentils. Under heat stress conditions, mineral concentrations among lentil genotypes varied from 6.0 to 8.8 mg/100 g for Fe and from 4.9 to 6.6 mg/100 g for Zn. Protein ranged from 21.9 to 24.3 g/100 g. Cooking time was significantly reduced due to high temperature treatment; the range was 3–11 min, while under no stress conditions, cooking time variation was from 5 to 14 min. Phytic acid variation was 0.5–1.2 g/100 g under no stress conditions, while under heat stress conditions, phytic acid ranged from 0.4 to 1.4 g/100 g. All genotypes had highly significant bioavailable Fe and moderately bioavailable Zn under no stress conditions. Whereas under heat stress conditions, Fe and Zn bioavailability was reduced due to increased phytic acid levels. Our results will greatly benefit the development of biofortified lentil cultivars for global breeding programs to generate promising genotypes with low phytic acid and phytic acid/micronutrient ratio to combat micronutrient malnutrition.

## Introduction

Lentil (*Lens culinaris* Medikus) is an ancient and early domesticated food legume crop originating from the Near East ~10,000 years ago (1). It has been part of the human diet since the early days of agriculture. Lentil belongs to the family Fabaceae and ranks fifth in global pulse production after common bean (*Phaseolus vulgaris* L.), dry pea (*Pisum sativum* L.), chickpea (*Cicer arietnum* L.), and cowpea (*Vigna unguiculate* L.) (2). The current global lentil production is 5.8 MT. Global lentil production is expected to increase due to the growing demand for plant-based protein and environment-friendly agriculture as it has a nitrogen-fixing capability to reduce energy costs in crop production (3).

Lentil is an integral part of the global food system with low inputs and provides a range of micronutrients (4). It is a rich protein source with 20–31% proteins, providing essential amino acids to the human body (5). Besides, lentil is also a good source of B-complex vitamins; it also contains a significant concentration of folate (6). Lentil seeds are relatively low in fat (<1%) and are an excellent supplement of the human diet in fiber and prebiotic carbohydrates (7, 8). Lentil provides a broad range of beneficial bioactive compounds such as polyphenols (9). Therefore, it is not surprising that lentil is the most desired legume in many lentil-producing regions worldwide because of the nutritional profiles. Besides their high nutritional value, lentils typically cook faster than other legumes because of their seed size and thin seed coat (10). Cooking time is an essential attribute in evaluating pulses, and the shorter cooking time is crucial to consumer preference as any fast cooking dish and end-use. Anti-nutritional factors (trypsin inhibitors, phytic acid, and tannins) reduce the digestibility of protein, carbohydrates, and micronutrient bioavailability in the human diet (11). Phytic acid is the primary storage form of phosphorus in legume seeds and plays a vital role in seed germination and seedling growth (12). Phytic acid is a potent chelator of positively charged cations including potassium (K), magnesium (Mg), calcium (Ca), iron (Fe), zinc (Zn), and mangenese (Mn) (13). Once consumed in human foods, phytic acid binds to these minerals in the intestinal tract to form mixed salts that are generally eliminated. This phenomenon poses a major risk of micronutrients deficiency (14).

Iron and Zinc deficiencies are the most widespread micronutrient deficiency globally. Micronutrient malnutrition is primarily due to the heavy reliance on staple food crop-based diets with low mineral bioavailability (15). Developing biofortified lentil varieties with high micronutrients bioavailability is a feasible approach to combat hidden hunger. However, several studies reported the genotypic and environmental factors affecting phytic acid and micronutrient concentrations in legume crops (16, 17). Furthermore, due to climate change, changes in temperature during the reproductive stage of legumes' development primarily affect their yield and nutritional value. Temperatures over 32°C during flowering and podding stages cause damage to reproductive organs, leading to significant losses in lentil grain yield (18). Outcomes from our previous study on the effect of heat stress on lentil seed quality revealed the adverse effect of rising temperature, at flowering and podding period, on seed quality components (19). Previous studies reported a significant reduction in Fe (18%), Zn (22%), and protein (14%) levels in lentils and observed an adequate genetic variation for Fe (0.5–10.9 mg/100 g), Zn (3.1–6.5 mg/100 g), and crude protein content (22.5–32.0 g/100 g) (19). In this regard, 36 lentil genotypes were selected based on their performance under heat stress conditions to test their response to grain yield and nutritional quality under controlled conditions. To achieve precision on the existing genetic variability of the nutritional quality of lentil with response to heat stress and the impact of rising temperature on the phytic acid and it effects on micronutrient bioavailability and cooking time in lentil seed, the present study was undertaken with the following objectives: (a) investigate high temperature effect on micronutrients, crude protein, phytic acid and cookability of selected genotypes, (b) to describe the variability in seed phytic acid concentration and estimate the bioavailability of Zn and Fe.

## Materials and Methods

### Plant Material

A set of 36 lentil genotypes was selected based on preliminary results of the quality traits and grain yield (19). Selected genotypes were also characterized by early flowering (<60 days after planting). Seeds were obtained from the International Center for Agricultural Research in the Dry Areas (ICARDA), Rabat, Morocco. Description of tested genotypes is presented in Supplementary Table S1. Plants were grown in plastic pots (15 cm in diameter and 20 cm in height) with 1.5 kg of a mixture of sandy loam soil and compost garden soil in a ratio of 2:2 (w/w). Six seeds were sown in each pot at 2 cm depth during the last week of December 2018 and reduced to four per pot after emergence. A randomized complete block design with two replicates was employed. To minimize the effects of variation in light profiles, the position of the pots was changed weekly during the experiment.

### Greenhouse Experiment

The experiment was conducted under two temperature regimes maintained in separate chambers designated no stressed and heat stressed. Plants under no stressed were grown with a mean day/night temperature of 22/12°C, whereas plants with heat stress were grown with a mean temperature of 25/14°C. For both chambers, the day length was 14 h (500 μmol m^−2^ s^−1^ light intensity). Relative humidity varied between 70 and 80% throughout the experimental period in both chambers. Heat stress was initiated with the pre-flowering stage by the increasing temperature at an average rate of 2°C per day to be the final temperature of 32°C at flowering stage. The plants remained under these conditions till maturity. Besides the heat stress, plants were watered and fertilized as per protocol developed at ICARDA, Morocco. At physiological maturity, plants were hand-harvested, threshed, and cleaned seeds were used to determine the grain yield and nutritional quality traits.

### Grain Yield Traits

Numbers of total pods, filled pods, unfilled pods, biological yield (g), grain yield (g), and hundred-seed weight (g) were determined for each treatment. All observations were taken on four plants per genotype for each replicate.

### Seed Size and Seed Shape Parameters

Lentil seeds were randomly selected to determine the hundred-seed weight and seed geometry by image analysis using a high-speed seed counting device (OptoAgri2, Optomachine, France). The OptoAgri2 combines a camera with a high resolution depending on the grain size, a laboratory balance, and software for image processing with an algorithm developed by OPTOmachines. This device measures seed area, perimeter, length, width, circularity, diameter, thickness, and rugosity. In addition to seed shape parameters, the software gives information about the hundred-seed weight.

### Mineral Concentration

Mineral concentration was measured using a previously published method (20). Five hundred milligrams from each sample were weighed into a glass tube and then 6 mL concentrated (70%) nitric acid (HNO_3_) was added. The tubes were then placed into a digestion block (QBlock series, Ontario, Canada) and heated for 60 min at 90°C. Three milliliters of 30% hydrogen peroxide (H_2_O_2_) were then added, and the samples were heated at 90°C for another 15 min, after which most of the residue was digested. Then, 3 mL of 6 M hydrochloric acid (HCl) was added to each sample. After cooling to ambient temperature, the volume was adjusted to 10 mL and then filtered. Fe and Zn concentrations were measured using inductively coupled plasma-optical emission spectroscopy (ICP-OES); (ICAP-7000 Duo, Thermo Fisher Scientific, France). Calibration curves for Fe and Zn were made using serial dilution from 0.1 to 10 mg L^−1^. Data validation was done using lab references and the NIST standard references.

### Protein Concentration

The total nitrogen content of the samples was determined using the Kjeldahl procedure (21). Samples were digested with a mixture of sulfuric acid, selenium, and salicylic acid. The mixture was then digested at 300°C for 5 h using a digestion block (QBlock series, Ontario, Canada). The filtrate was treated with 5.5 ml of the buffer solution, 4.4 ml of sodium nitroprusside, and 2 ml of sodium hypochlorite. The mixture is placed in the dark at 37°C for 15 min. The sample's absorbance was measured at 650 nm. The percentage of protein content was calculated by multiplying the Kjeldahl nitrogen by the conversion factor of 6.25.

### Phytic Acid Concentration

A Megazyme kit was used to measure the phytic acid concentration (22). One gram of ground lentil seed was digested with 20 mL of HCl (0.66 M) solution in 50 ml flacon tubes and placed in a shaker overnight (15 h) at room temperature. The day after, 1 mL of the extract was subjected to several enzymatic reactions to release inorganic phosphorus (Pi) from phytic acid. The inorganic phosphorus (Pi) was then reacted with ammonium molybdate to form 12-molybdophosphoric acid, which is subsequently reduced under acidic conditions to molybdenum blue. The amount of molybdenum blue formed in this reaction is proportional to the amount of Pi present in the original sample and hence to phytic acid. A UV-Visible spectrophotometer (T80 series, pg instruments, UK) was used to measure the absorbance of molybdenum blue at 655 nm. The phosphorus solutions were prepared as described by the Megazyme manual, using a standard phosphorus solution (24 mL, 50 μg/mL), and treated as samples for the colorimetric determination of phosphorus.

The inorganic phosphorus (Pi) is quantified as phosphorus from the generated calibration curve. At the same time, the calculation of phytic acid content assumes that the amount of phosphorus measured is exclusively released from phytic acid and comprises 28.2% of phytic acid.


$$\begin{array}{l} \text{Phytic acid}=\frac{\text{Phosphorus}\left[\text{g}/100\text{g}\right]}{0.282} \end{array}$$

### Phytic Acid/Iron and Phytic Acid/Zinc Molar Ratios

The molar ratios phytic acid/iron and phytic acid/zinc were calculated according to the following equation:


$$\begin{array}{l} \text{PA}/\text{MN ratio}=\frac{\text{PA}/\text{M}{\text{W}}_{\text{AP}}}{\text{MN}/\text{M}{\text{W}}_{\text{MN}}} \end{array}$$

where: PA is phytate acid content; MW_PA_ is PA molecular weight (660.04 g mol^−1^); MN is micronutrient content (zinc or iron); MW_MN_ is micronutrient molecular weight (Zn = 65.4 g mol^−1^; Fe = 55.85 g mol^−1^).

### Cooking Time

The automated Mattson Cooker (Canadian Grain Commission, Canada) consisted of a cooking rack with 25 perforated depressions and 25 weighted plungers (80 g) of 2 mm tip diameter was used to measure lentil seed cooking time. Before cooking, the samples (2 g of lentil) were soaked in 50 mL of distilled water and maintained at room temperature (22 ± 2°C) for 12 h. Twenty-five soaked seeds were randomly selected and then placed in each of the 25 perforations of the rack. The tip of each plunger was rested on top of the seed. The loaded Mattson Cooker was then placed into a 2 L beaker containing 1.5 L of boiling distilled water, and the beaker was covered to reduce evaporation. The apparatus was maintained over direct heat using a hot plate. During testing, the hot plate was set at 390°C to maintain water boiling. Lentil seeds are considered cooked when the plungers penetrate the seeds and touch the sensor on the fourth rack (sensor assembly); the Easy CT program automatically recorded cooking time for each seed. Cooking time is defined as the number of minutes required for 80% of lentil seeds to be pierced.

### Statistical Analysis

Summary data was reported as range and mean values ± standard deviation. The coefficient and the *p*-value of the correlations were calculated using Pearson's correlation. Analysis of variance (ANOVA) was used to test the effects of treatments, genotypes, and genotype x treatment interaction on the measured traits. Principal component analysis (PCA) was performed using the *Factoextra* (23) and *FactoMineR* (24) packages in R version 4.1.0 and RStudio version 1. 3.1093. Hierarchical cluster analysis was conducted using Ward's squared Euclidean distance method with the *dendextend* R package (25).

## Results

The combined analysis of variance showed significant genotypic differences for all evaluated traits of 36 tested genotypes (Supplementary Table S2). The effect of high temperature was also significant (*p* < 0.001) for all measured traits, except for the number of unfilled pods, seed eccentricity, and Feret's diameter.

Similarly, genotype × treatment interaction was highly significant (*p* < 0.001) for all investigated traits, excluding seed width, area, perimeter, equivalent diameter, and rugosity.

### Heat Stress Effect on Grain Yield and Yield-Related Traits

Grain yield decreased substantially (36.6%) by exposure to heat compared to the no stress plants, which had a mean of 2.3 g plant^−1^. The biological yield was higher for the no stress plants; the range was 6.1–16.4 g, with a mean of 11.3 g. Whereas, biological yield in plants exposed to high temperature showed a marked reduction of 38.2% (Table 1). Under high temperature conditions, the total number of pods plant^−1^ was particularly low compared with no stress conditions, with a mean of 61.1. Similarly, heat stress reduced the total number of filled pods plant^−1^ by 30.9% and relatively increased the number of unfilled pods plant^−1^.

**Table 1:** Range and mean ± SD of grain yield, yield related traits, seed size and shape parameters under normal and heat stress conditions.

**Trait**	**No stress**		**Heat stress**	
	**Range**	**Mean ±SD**	**Range**	**Mean ±SD**
GY	0.9-3.8	2.3, 0.7	0.9-2.1	1.4, 0.3
BY	6.1-16.4	11.3, 2.5	4.4-11.7	7.0, 1.7
FPP	14.8-82.4	50.3, 16.6	14.8-54.4	34.8, 9.0
UPP	3.5-28.6	10.8, 4.2	2.3-19.3	11.6, 5.2
TPP	30.3-105.5	61.1, 18.7	17.9−68.3	46.4, 11.7
HSW	0.8-2.9	1.3, 0.5	0.7-1.9	0.9, 0.3
SL	3.6-6.1	4.8, 0.6	3.4-6.6	5.0, 0.7
SW	3.3-5.7	4.5, 0.6	4.7-6.2	4.7, 0.6
SA	9.3-26.7	16.9, 4.4	8.4-31.6	18.5, 4.9
SP	13.3-22.9	18.0, 2.4	12.7-25.0	18.8, 2.6
SD	3.4-5.8	4.6, 0.6	3.2-6.3	4.8, 0.6
SE	0.3-0.4	0.3, 0.0	0.3-0.4	0.33, 0.0
SFD	1.0-1.1	1.1, 0.0	1.0-1.1	1.1, 0.0
SC	1.0-0.0	1.0, 0.0	1.0-1.0	1.0, 0.0
ST	0.8-0.8	0.8, 0.0	0.8-0.8	0.8, 0.0
SR	0.2-0.3	0.2, 0.0	0.2-0.3	0.2, 0.0

*GY, Grain yield (g); BY, Biological yield (g); FPP, Filled pods plant^−1^; UPP, Unfilled pods per plant^−1^; TPP, Total pods plant-1; HSW, hundred-seed weight; SL, Seed length (mm); SW, Seed width (mm); SA, Seed area (mm2); SP, Seed perimeter (mm); SD, Seed diameter (mm); SE, Seed eccentricity (mm); SFD, Feret's diameter; SC, Seed circularity; ST, Seed thickness; SR, Seed rugosity*.

### Heat Stress Effect on Seed Size and Shape Parameters

Heat application resulted in a slight increase in seed length, width, area, perimeter, and equivalent diameter; the mean was 5 mm, 4.7 mm, 18.5 mm^2^, 18.8 mm, and 4.8 mm, respectively. However, the mean was 4.8 mm, 4.5 mm, 16.9 mm^2^, 17.0 mm, and 4.6 mm in normal conditions, respectively. In contrast, heat stress has no impact on seed eccentricity, Feret's diameter, thickness, and rugosity. Compared to normal conditions, the hundred-seed weight decreased by 27.2% due to heat stress (Table 1).

### Heat Stress Effect on Nutritonal Quality

The mean Fe concentration declined in genotypes exposed to heat stress compared to no stress conditions. The range varied from 6.0 to 8.8 mg/100 g, with a mean of 7.2 mg/100 g. Whereas, under no stress conditions, the mean was 8.6 mg/100 g. Comparatively, Zn concentration was negatively influenced in response to high temperature; the values range from 5.0 to 6.6 mg/100 g with a mean of 5.4 mg/100 g. As compared to no stress conditions, high temperature caused a reduction in protein concentration. The maximum value for protein under no stress conditions was 27.4 g/100 g, while in heat stress, it reduced to 24.3 g/100 g. High temperature induced an increase in total phytic acid, which varied from 0.4 to 1.4 g/100 g; the mean was 1.0 g/100 g. While, under no stress conditions, total phytic acid varied from 0.5 to 1.2 g/100 g, with a mean of 0.9 mg/g. As for cooking time, the values ranged from 5 to 14 min; the mean cooking time was 8.1 min under no stress conditions. In contrast, cooking time showed a significant reduction (28.3%) under high temperature treatment, with a mean of 5.8 min (Table 2).

**Table 2:** Range and mean ± SD of seed quality traits and cooking time under normal and heat stress conditions.

**Trait**	**No stress**		**Heat stress**	
	**Range**	**Mean ±SD**	**Range**	**Mean ±SD**
Fe	7.0–10.0	8.6, 0.8	6.0-8.8	7.2, 0.7
Zn	5.4–7.7	6.5, 0.6	5.0-6.6	5.4, 1.1
CP	20.3–27.4	23.6, 2.2	20.3-24.3	21.9, 1.0
CT	5–14	8.1, 2.6	3-11	5.8, 2.0
PA	0.5–1.2	0.9, 0.2	0.4-1.4	1.0, 0.2
PA/Fe	4–12	8.6, 2.0	7-17	11.3, 2.2
PA/Zn	7–18	13.4, 2.9	11-26	16.8, 3.1

*Fe, Iron content (mg/100 g); Zn, Zinc content (mg/100 g); CP, Crude protein (g/100 g); CT, Cooking time (min); PA, Phytic acid (g/100 g); PA/Fe, Phytic acid/Iron ratio; PA/Zn, Phytic acid/Zinc ratio*.

### Heat Stress Effect on Micronutrient Bioavailability

The mean of both phytic acid/micronutrients (Fe and Zn) molar ratios showed reduced value compared to heat stress conditions. The high temperature increased PA/Fe and Zn molar ratios the mean was 11.3. and 16.8 (Table 3). Under no stress conditions, all studied lentil genotypes had a phytic acid/iron molar ratio above the critical value of 14, that estimates a good Fe bioavailability. Whereas, ILL 224, ILL950, ILL 4881, ILL 5968, ILL 5416, ILL 4804, ILL 597, ILL 6281, Zaaria, ILL 5261, and ILL 6644 had a phytic acid/zinc molar ratio above the critical values of 15. On the other hand, under heat stress conditions, the phytic acid/iron molar ratio was above the threshold of 14 for ILL 5505 ILL 2230 and ILL 956. As for phytic acid/zinc ratio, there was a shift of 13 genotypes from moderately to low bioavailable Zn and two genotypes from low to moderately bioavailable Zn, while the rest maintain the same category as in the normal conditions (Table 3).

**Table 3:** Iron and zinc bioavailability of tested genotypes under no stress and heat stress conditions.

**Micronutrient**	**Level**	**No stress**	**Heat stress**
Iron bioavailability	High	ILL 494, ILL 2181, LSI88, ILL 5509, ILL 1959, ILL 4345, Chakkouf, ILL 956, ILL 5595, ILL 3517, ILL 7084, ILL 4841, ILL 4738, ILL 6528, Bichette, ILL 619, ILL 624, ILL 918, ILL 5505, ILL 4471, ILL 6493, ILL 2230, ILL 257, ILL 6870, ILL 4791, ILL 224, ILL 950, ILL 4881, ILL 5968, ILL 5416, ILL 4804, ILL 597, ILL 6281, Zaaria, ILL 5261, ILL 6644	ILL 494, ILL 2181, LSI88, ILL 5509, ILL 1959, ILL 4345, Chakkouf, ILL 5595, ILL 3517, ILL 7084, ILL 4841, ILL 4738, ILL 6528, Bichette, ILL 619, ILL 624, ILL 918, ILL 4471, ILL 6493, ILL 257, ILL 6870, ILL 4791, ILL 224, ILL 950, ILL 4881, ILL 5968, ILL 5416, ILL 4804, ILL 597, ILL 6281, Zaaria, ILL 5261, ILL 6644
	Low	None	ILL 5505, ILL 956, ILL 2230
Zinc bioavailability	Moderate	ILL 494, ILL 2181, LSI88, ILL 5509, ILL 1959, ILL 4345, Chakkouf, ILL 956, ILL 5595, ILL 3517, ILL 7084, ILL 4841, ILL 4738, ILL 6528, Bichette, ILL 619, ILL 624, ILL 918, ILL 5505, ILL 4471, ILL 6493, ILL 2230, ILL 257, ILL 6870, ILL 4791	ILL 5968, ILL 4841, Chakkouf, ILL 5261, Bichette, ILL 4881, Zaaria, ILL 494, ILL 2181, ILL 6493, ILL 597
	Low	ILL 224, ILL 950, ILL 4881, ILL 5968, ILL 5416, ILL 4804, ILL 597, ILL 6281, Zaaria, ILL 5261, ILL 6644	ILL 4791, ILL 257, ILL 6528, ILL 4345, ILL 619, ILL 6870, ILL 624, ILL 950, ILL 3517, ILL918, LSI88, ILL 4804, ILL 224, ILL 6644, ILL 7084, ILL 6281, ILL 1959, ILL 4471, ILL 5416, ILL 5595, ILL 5509, ILL 2230, ILL 4738, ILL 956, ILL 5505

*(<5), High zinc bioavailability; (5–15), Moderate zinc bioavailability; (>15) Low zinc bioavailability. (<14) Moderate iron bioavailability; (>14), Low iron bioavailability*.

### Association Between Seed Yield, Seed Shape and Nutritional Quality Traits

The correlation of grain yield with total number of pods and filled pods was positive and highly significant in both environments. Grain yield showed a highly significant positive correlation with biological yield under no stress conditions (*r* = 0.62; *p* < 0.001), while under heat stress treatment, this correlation was positive but not significant (Supplementary Table S3). Another positive and highly significant correlation, in both no stress and heat stress treatments, was observed between total number of filled pods and grain yield (*r* = 0.79; *p* < 0.001) and (*r* = 0.66; *p* < 0.001), respectively. The correlation between seed shape features and both grain yield and 100-seed weight of was statistically non-significant under both no stress and heat stress conditions. However, seed geometric features showed highly significant correlations among them, these correlations followed the same pattern under both environments with the exception of few parameters. Seed circularity showed a significant negative correlation with seed length (*r* = −0.37; *p* < 0.05), width (*r* = −0. 43; *p* < 0.01), area (*r* = −0. 37; *p* < 0.05), perimeter (*r* = −0. 40; *p* < 0.05) and equivalent diameter (*r* = −0. 41; *p* < 0.05) under high temperature treatment, whereas, under normal conditions these correlations were negative but remained non-significant. Under no stress conditions, seed thickness was negatively correlated with seed circularity (*r* = −0.64; *p* < 0.001) and Feret's diameter (*r* = −0. 36; *p* < 0.05), while under heat stress conditions seed thickness showed a significant negative correlation only with seed circularity (*r* = −0. 43; *p* < 0.01). On the other hand, all seed shape parameters showed no correlation with hundred-seed weight (Supplementary Table S3).

**Figure 1:**
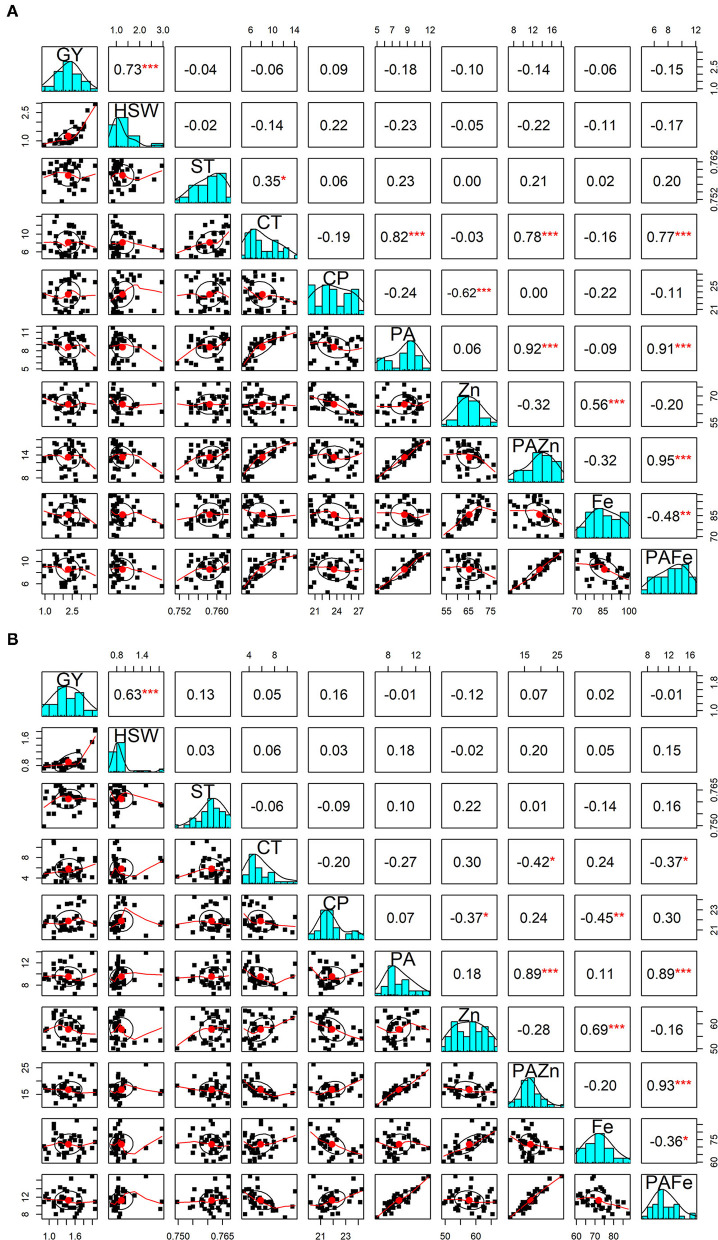
Pearson correlations between grain yield and seed qaulity traits. The diagonal panels show histograms for each trait. The lower and upper triangular panels, respectively, show scatter plots and Pearson correlation coefficients between grain yield and seed quality traits under no stress **(A)** and heat stress **(B)** conditions. GY, Grain yield (g); HSW, hundred-seed weight (g); ST, Seed thickness; CT, Cooking time (min); CP, Crude protein (g/100 g); PA, Phytic acid (g/100 g); Zn, Zinc content (mg/100 g); PA/Zn, Phytic acid/Zinc ratio; Fe, Iron content (mg/100 g); PA/Fe, Phytic acid/Iron ratio. *, **, and *** indicate significance at 0.05, 0.01, and 0.001 probability levels, respectively.

The correlations between grain yield and hundred-seed weight with different quality traits were also computed under both no stress and heat stress conditions (Figure 1). Interestingly, almost all the correlations were found to be statistically non-significant. There was no significant correlation between seed geometric parameters and seed quality traits under high temperature treatment, while under normal conditions, cooking time was found to be negatively correlated with seed circularity (*r* = −0.33; *p* < 0.05) and positively correlated with seed thickness (*r* = 0.35; *p* < 0.05). Among the nutritional quality traits, a significant positive association was identified between zinc and iron content under both no stress and heat stress conditions (*r* = 0.56 and *r* = 0.69; *p* < 0.001, respectively). In contrast, a significant negative correlation was detected between zinc and protein under both conditions. The correlation between iron and protein was negative and significant under heat stress treatment (*r* = −0.45; *p* < 0.01), whereas iron content did not appear to be significantly correlated with protein content under normal conditions. A highly significant negative association was obtained between phytic acid and cooking time under no stress conditions (*r* = 0.82, *p* < 0.001), while under heat stress conditions the association was negative but not significant. Cooking time and phytic acid showed no significant correlation with grain yield and other quality traits under normal and high temperature conditions. When looking at phytic acid/micronutrients ratio, PA/Zn showed a highly positive and significant correlation between cooking time (*r* = 0.78; *p* < 0.001), phytic acid (*r* = 0.91; *p* < 0.001) and PA/Fe ratio (*r* = 0.95; *p* < 0.001) under no stress conditions. On the other hand, under heat stress conditions, this ratio showed a negative and significant correlation with cooking time (*r* = −0.52; *p* < 0.01) and a highly positive significant correlation with phytic acid (*r* = 0.90; *p* < 0.001) and PA/Fe (*r* = 0.94; *p* < 0.001).

With respect to PA/Fe ratio, a highly positive and significant correlation was obtained with cooking time (*r* = 0.77; *p* < 0.001) and phytic acid (*r* = 0.91; *p* < 0.001) under no stress conditions. Whereas, under high temperature conditions, PA/Fe ratio revealed a highly significant positive association with phytic acid (*r* = 0.88; *p* < 0.001) and a significant negative correlation with cooking time (*r* = −0.51; *p* < 0.01). In contrast, PA/Fe ratio showed significant negative correlation with iron content under both conditions.

### Principal Component Analysis

**Figure 2:**
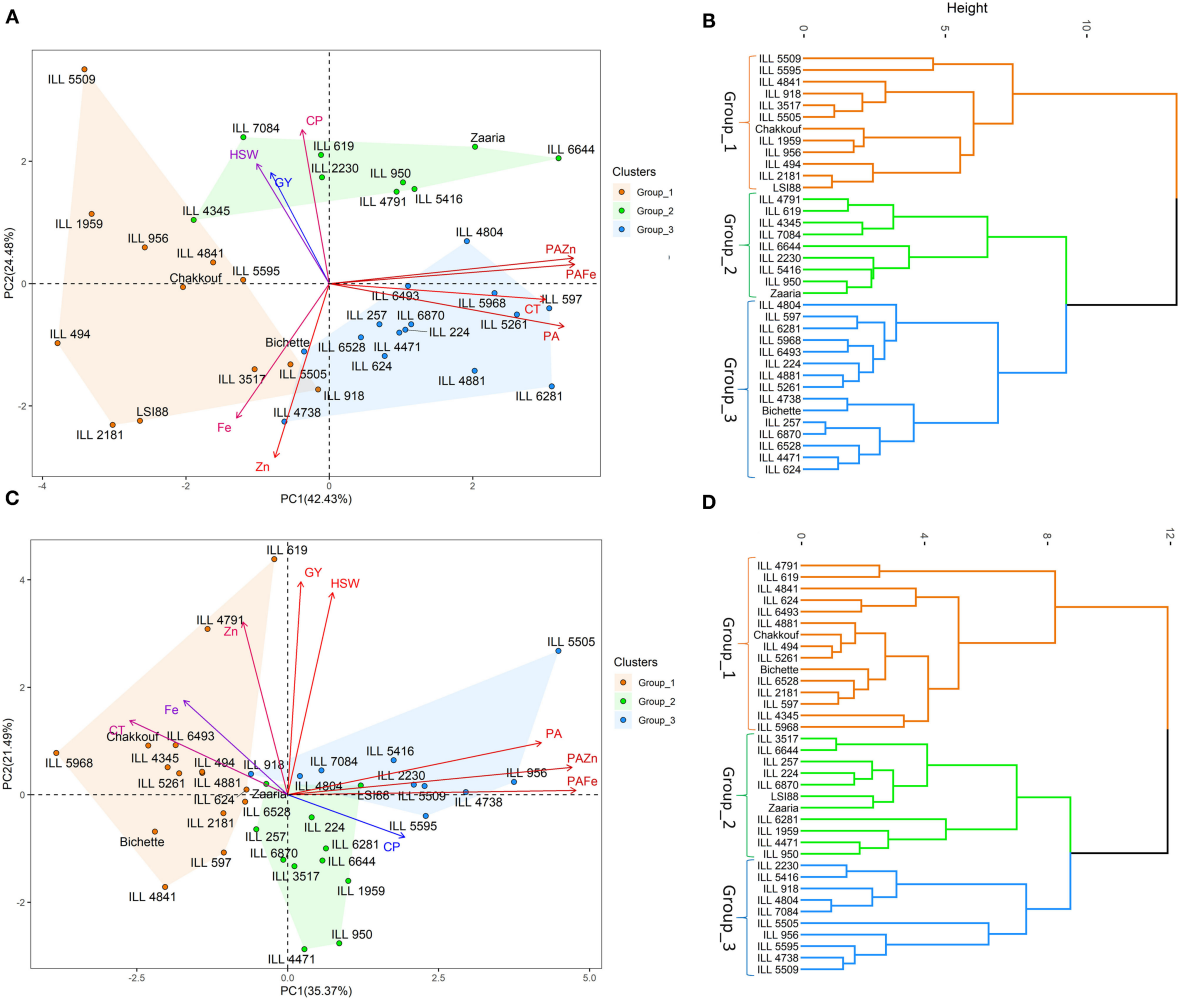
Principal component analysis, hierarchical clustering and various traits contributing to the variability under no stress **(A,B)** and heat stress **(C,D)** conditions. GY, Grain yield (g); HSW, hundred-seed weight (g); CT, Cooking time (min); CP, Crude protein (g/100 g); PA, Phytic acid (g/100 g); Zn, Zinc content (mg/100 g); PA/Zn, Phytic acid/Zinc ratio; Fe, Iron content (mg/100 g); PA/Fe, Phytic acid/Iron ratio.

Under no stress conditions, the first two principal components explained 66.8% of the total variability (Table 4). PC1 and PC2 accounted for 42.4 and 24.5% of the total variation, while PC3 explained 16.2% of the variation. Cooking time, phytic acid and phytic acid/iron and zinc ratios showed the highest contribution to the first principal component (PC1). Crude protein, iron and zinc contents contributed the most for PC2, whereas grain yield and hundred-seed weight were the most contributing traits to PC3 (Figure 2). On the other hand, under heat stress conditions, the first two axes of the PC explained 56.9% of the total variation. PC1 accounted for 35.4% of the total variation, and PC2 and PC3 explained 21.5 and 56.9% of the total variation. The PC1 variation was mainly due to phytic acid and phytic acid/ iron and zinc ratios. The variation in PC2 was associated with grain yield, hundred-seed weight, and zinc content, while the variation in PC3 was due to iron and crude protein contents.

**Table 4:** Percentage of contribution of different traits to the three major principal components with percentage variation under the different treatments.

**Traits**	**No stress**			**Heat stress**		
	**PC1**	**PC2**	**PC3**	**PC1**	**PC2**	**PC3**
GY	1.4	12.1	37.5	0.1	33.4	13.6
HSW	2.2	14.1	32.1	0.7	30.1	5
CT	19.2	0.2	4.2	8.9	4.1	0.5
CP	0.3	23.3	9.5	4.9	1.3	31.5
PA	22.7	1.8	2.7	22.8	2	14
Zn	1.2	29.7	10.5	0.7	22	1.1
PA/Zn	24.6	0.6	0.04	28.7	0.6	1.9
Fe	3.6	17.8	3.03	3.8	6.6	32
PA/Fe	24.8	0.4	0.3	29.5	0.01	0.5
Percentage variation (%)	42.4	24.5	16.2	35.4	21.5	56.9

*GY, Grain yield (g); HSW, hundred-seed weight (g); CT, Cooking time (min); CP, Crude protein (g/100 g); PA, Phytic acid (g/100 g); Zn, Zinc content (mg/100 g); PA/Zn, Phytic acid/Zinc ratio; Fe, Iron content (mg/100 g); PA/Fe, Phytic acid/Iron ratio*.

Hierarchical cluster analysis clearly classified tested genotypes into three distinct groups; the mean values of each group are listed in Table 5. Under no stress conditions, group_1 was characterized by high grain yield, hundred-seed weight and iron and zinc contents; the mean was 2.6 g, 1.5 g and 9.2 mg/100 g, respectively. Group_1 was also characterized by a short cooking time and a low phytic acid. The second group contained genotypes with high grain yield, hundred-seed weight and protein content, the mean was 2.5 g, 1.3 g and 25.6 g/100 g, respectively. Genotypes belonging to group_3 had a high level of phytic acid, long cooking time and a low bioavailable zinc. Under heat stress conditions, cluster analysis also revealed the existance of three groups. Genotypes falling into group_1 showed high grain yield and phytic acid and long cooking time,While group_2 was characteriezed by high protein content and low bioavailable zinc content. Group_3 was distinguished by a very high phytic acid level and a very low bioavailable iron and zinc content (Table 5).

**Table 5:** Mean ± SD of seed quality traits, cooking time and phytic acid/ micronutrient ratio of the three clusters under no stress and heat stress conditions.

**Trait**	**Group_1**		**Group_2**		**Group_3**	
	**Normal**	**Heat**	**Normal**	**Heat**	**Normal**	**Heat**
GY	2.6, 0.6	1.6, 0.3	2.5, 0.4	1.3, 0.3	1.8, 0.6	1.5, 0.3
HSW	1.5, 0.7	0.9, 0.4	1.3, 0.4	0.9, 0.2	1.1, 0.2	0.9, 0.2
CT	6.1, 1.0	6.8, 2.0	8.0, 2.3	5.6, 2.1	9.8, 2.4	4.5, 0.8
CP	23.1, 2.4	21.4, 0.5	25.6, 1.1	22.4, 0.9	22.5, 1.4	22.1, 1.6
PA	0.7, 0.2	0.8, 0.1	0.8, 0.2	0.9, 0.1	1.0, 0.1	1.2, 0.1
Zn	6.9, 0.6	5.7, 0.8	5.9, 0.3	4.5, 1.3	6.7, 0.3	5.8, 0.5
PA/Zn	10.4, 2.2	14.3, 1.6	14.6, 2.5	16.9, 1.2	15.0, 1.6	20.3, 2.9
Fe	9.2, 07	7.5, 0.6	7.7, 0.6	6.7, 0.6	8.6, 0.6	7.3, 0.6
PA/Fe	6.5, 1.4	9.4, 1.0	9.4, 1.8	11.7, 0.9	9.8, 1.2	13.7, 2.0

*GY, Grain yield (g); HSW, hundred-seed weight (g); CT, Cooking time (min); CP, Crude protein (g/100 g); PA, Phytic acid (g/100 g); Zn, Zinc content (mg/100 g); PA/Zn, Phytic acid/Zinc ratio; Fe, Iron content (mg/100 g); PA/Fe, Phytic acid/Iron ratio*.

## Discussion

Our results indicated that temperature beyond 30°C has a detrimental impact on seed yield and nutritional quality of investigated lentil genotypes. All genotypes were to some extent adversely affected by heat stress regarding biological yield, grain yield, and hundred-seed weight, which was in accordance with our previous study where heat stress decreased biological yield, grain yield and hundred-seed weight by 42.2, 53.5, and 26.8%, respectively (19). Our observations in this regard were similar to those in many studies. Sehgal et al. (26) revealed that application of heat stress during the reproductive stage reduced seed weight by 65–68% in two heat-tolerant genotypes. Supporting results were recently published, where terminal heat at the reproductive stage adversely affected both biological yield and grain yield but not hundred-seed weight (18). Similarly, heat stress during the grain filling period significantly reduced seed yield of chickpea genotypes (27) and common bean (28). Reduction in grain yield can be explained by the fact that high temperature during the reproductive stage negatively affects flower production (29). Failure of pod set can be explained by the negative impact of high temperature on pollen germination, pollen viability, pollen tube growth through the style (30, 31). In addition, our results indicated that the number of filled pod was adversely reduced under high temperature treatment, which is in good agreement with the above mentioned findings.

Seed size determination in lentils has historically relied on measuring the hundred or thousand seed weight (32). However, this method cannot give an indication of the actual seed shape parameters. Therefore, we used a computer-assisted image analysis method to determine both seed weight and seed shape. Our results showed that high temperature treatment induced slight changes in seed shape parameters. Seed length, width, perimeter and equivalent diameter showed a slight increase, while seed area was noted as moderately influenced character under heat stress. On the other hand, seed eccentricity, Feret's diameter, circularity and rugosity were not affected by heat stress. Little information is available in this regard, however, studies on other crops have revealed that seed shape related traits, such as seed width, seed length, and seed weight, are complex quantitative traits governed by polygenes and highly influenced by environmental conditions (33). Eşref and Nazmi (34) investigated the effect of different levels of moisture content on certain physical properties of the yellow lentil, results from this study showed that an increase in moisture content level led to an increase in seed area, diameter, thickness and diameter. On the other hand, correlation analysis in our study revealed no significant association between hundred-seed weight and seed shape parameters under both stress and no stress conditions. In contrast to our findings, Saha et al. (35) demonstrated that the hundred-seed weight of lentil had a higher positive correlation with seed diameter (*r* = 0.88; *p* < 0.01). Wang et al. (36) showed that the hundred-seed weight showed a high significant correlation with seed length, width and thickness in soybean, whereas, no correlation was detected between the hundred-seed weight and length/width ratio, length/thickness ratio, and width/thickness ratio.

The effect of high temperature on seed quality during reproductive stage was observed in many crops. Our previous findings have also highlighted the impact of heat stress on iron, zinc and crude protein (19). Similarly, in the present study, high temperature resulted in a significant reduction in both micronutrients. It has been reported that a moderately high temperature reduced the rate of nutrient uptake by roots in many crops (37). Therefore, increasing temperature due to global warming will likely have overall negative effects on plant nutrient relations, which will surely contribute to decreases in micronutrients level of lentil seed. Supporting results reported that high temperature resulted in a substantial decline in iron and zinc contents in lentil (38). High temperature also had a clear effect on protein content of tested genotypes. Recently, El Haddad et al. (39) reported that high temperature significantly decreased protein content, iron and zinc concentration in lentil by 15, 14, and 15%, respectively. Reduction in the rate of protein content accumulation and storage in legume seeds can be explained by inhibition of protein synthesis processes due to heat stress (29). Supporting studies have shown similar results in lentil (38), chickpea (40), soybean (41) and bean (42).

Due to the presence of a large genetic variability coupled with high heritability estimates (43, 44), lentil holds a great potential toward genetic biofortification for increased Fe and Zn content. However, many studies reported that iron and zinc absorption from diets based on legumes was found to be low (45). A major constraint to iron and zinc intake is the presence of antinutrients like phytic acid (46). In this study, tested genotypes were evaluated for their level of phytic acid under normal and high temperature conditions. Our results in this regard showed significant variation for phytic acid among studied genotypes and between treatments. Under no stress conditions, the variation for phytic acid ranged from 0.5 to 1.2 g/100 g with a mean of 0.9 g/100 g. Thavarajah et al. (20) reported a smaller range of variation (0.3–0.4 g/100 g) for phytic acid in lentil genotypes from three different market classes grown at two locations in Saskatchewan, Canada. Whereas, the Spanish brown cultivar Pardina grown in Spain, had higher level of total seed phytic acid concentration (1.2/100 g), that exceeded the mean value of phytic acid in our study. Similarly, Paul and Shang (47) found more similar mean value in 17 lentil genotypes grown in Bangladesh (0.7/100 g). On the other hand, high temperature treatment resulted in an increase of phytic acid level of tested genotypes. In agreement with our findings, Thavarajah et al. (48) indicated that phytic acid concentrations in lentil seeds were significantly higher in the rising temperature regime. Similar results were also found in common bean (49). Accumulation of phytic acid under stress conditions is likely related to its function in limiting oxidative stress in legumes (50). Accordingly, phytic acid has been reported to accumulate in chickpea seeds in response to drought stress (51).

To estimate the potential bioavailability of iron and zinc in lentil seeds, phytic acid/micronutrients molar ratios were calculated. It is considered that a higher molar ratio can decline mineral bioavailability and vice versa. According to Gibson (52), phytic acid/zinc molar ratios <5, between 5 and 15, and >15 have been associated with high, moderate and low zinc bioavailability. Whereas, phytic acid/iron molar ratio >14 can be a cause of iron deficiency (53, 54). In the present study, the calculated PA/Fe mean molar ratio under normal conditions was 8.6 with all genotypes below 14. This suggests that iron absorption might not be significantly impaired by phytic acid present in these genotypes. However, the mean PA/Fe molar ratio increased under high temperature treatment. Genotypes ILL 2230, ILL 5505, and ILL 956 showed a ratio above the critical value (13), and likely have a low bioavailable iron in their seeds. Regarding phytic acid/zinc ratio, twenty-five of the tested genotypes have a ratio below 15 and fell into moderate zinc bioavailability category, while other genotypes were low in bioavailable zinc. On the other hand, the accumulation of phytic acid due to rising temperature resulted in a shift of the PA/Zn molar ratio of the majority of genotypes from moderate to low bioavailability category. Collectively, these results showed that zinc content is the most affected micronutrient in terms of bioavailability, which can be explained by the fact that zinc forms the most stable complex with phytate contributing to a low bioavailability of zinc (55). Phytic acid/micronutients ratios in our study were less than those previously recorded levels in chickpea (56), faba bean (57), dry pea and kidney bean as well as in common bean, black gram and mung bean (58).

Our study showed a high genotypic variability for cooking time under both conditions. The mean cooking time under no stress conditions was 8 min, which was less than those previously reported by other authors (59, 60), while, the mean cooking time of genotypes under high temperature treatment was lower than the normal conditions. There is no available information related to the impact of heat stress on cooking time of lentil. However, it has been documented that cooking time is affected by many factors including climatic conditions, harvesting processes and seed storage conditions (61, 62). Furthermore, cooking time is mainly influenced by mineral nutrition, an adequate soil supply of phosphorus is essential to enhance lentil cooking quality (63). Correlation analysis in our study showed that cooking time was positively correlated with phytic acid under normal conditions. Supporting results were previously published, where high seed phytic acid content was related to high cookability in lentil (64, 65). It is noteworthy to mention that a decrease in phytic acid resulted in hard-to-cook phenomenon of many legume crops, including lentil. Galiotou-Panayotou et al. (66) stated that a decrease in phytic acid from 10.4 to 7.2 mg g^−1^ induced a 42% increase in hardness. This suggests that low cookability of lentil seed grown under high temperature was due to the high level of phytic acid.Cooking time showed no significant correlation with 100-seed weight under both conditions, which was in accordance with finding reported by Ninou et al. (67). However, significant positive correlation has been reported in lentil (68) and common bean (69). Correlation analysis also showed that cooking time was positively correlated with seed thickness under normal conditions. In contrast, a non-significant correlation between cooking time and seed thickness has been demonstrated in lentil (67). Moreover, this seed shape parameter also showed non-significant correlation with cooking time under high temperature treatment. It should be pointed out that seed thickness has been reported to be largely influenced by environmental conditions during seed development (70). However, in our study seed thickness showed no reduction under high temperature treatment. From the very few studies reported here on factors influencing cooking time in lentil, our results suggests that a decrease in cooking time is connected with an increase in seed phytic acid level rather than its shape parameters.

In this study, Principal Component Analysis was used to define important traits contributing to the total variation in 36 lentil genotypes. The variation was mainly explained by the nutritional quality traits and grain yield, which were in accordance with our previous findings (19). In addition, Hierarchical Cluster Analysis was used to classify these genotypes based on similar nutritional quality traits and grain yield under both normal and stress conditions. Among the three identified groups, group_1 had the highest grain yield, hundred seed weight and micronutrients level. This group was also characterized by a highly bioavailable iron and zinc, a short cooking time and a low phytic acid. Genotypes belonging to this group could be useful for biofortification programs. Under heat stress conditions, genotypes belonging to group_1 showed high grain yield and phytic acid and long cooking time. These genotypes were able to defend themselves from heat stress by increasing the phytic acid level in their seeds. The use of multivariate analysis coupled with hierarchical cluster analysis provides an effective means for identifying the components that determine the variation when considering several traits simultaneously, and for grouping genotypes with similar traits. However, these findings can be further verified in a genotype x environment study to assess their nutritional quality and yield performance across different environmental conditions.

## Conclusion

The current study has shown differences in micronutrients (Fe and Zn), protein content, phytic acid and cooking time, along with the evaluation of their responses to high temperature treatment. Our findings revealed that high temeprature will not only decrease the nutritional quality of lentil seed, but will also incease antinutrients like phytic acid, leading to a decrease in cooking time of lentil. In this regard, we have focused on the effect of phytic acid because of its major influence on iron and zinc bioavailability. Our results showed that the phytic acid/micronutrients molar ratios were somehow smaller under no stress conditions, while these ratios were higher under heat stress especially for phytic acid/zinc ratio, indicating that the estimated bioavailability of these micronutrients is low and is mainly affected by the phytic acid. However, a total of 11 promising genotypes have been identifid under high temperature conditions, namely ILL 5968, ILL 4841, Chakkouf, ILL 5261, Bichette, ILL 4881, Zaaria, ILL 494, ILL 2181, ILL 6493 and ILL 597. These genotypes were characteriased by high yielding short cooking time, high iron and moderate zinc bioavailability. Our findings could be useful for generating varieties with appropriate levels of phytic acid and micronutrients, which can contribute in mitigating micronutrients malnutrition.

## Data Availability Statement

The original contributions presented in the study are included in the article/Supplementary Material, further inquiries can be directed to the corresponding author/s.

## Author Contributions

HC performed the research work and prepared first draft of the manuscript. HC, KA, and KH raised experiments in the greenhouse and recorded phenotyping data. HC and AE-B analyzed the seed samples for grain micronutrients in cereal and legumes quality laboratory. HC and NE performed statistical analysis of data. SK planned and supervised research activity. AS, FM, DT, and SK contributed in the final draft of the manuscript. All authors reviewed and approved the final version of the manuscript.

## Funding

The partial funding support provided by the CGIAR Research Program on Grain Legumes and Dryland Cereals (GLDC) and Government of India is duly acknowledged.

## Conflict of Interest

The authors declare that the research was conducted in the absence of any commercial or financial relationships that could be construed as a potential conflict of interest.

## Publisher's Note

All claims expressed in this article are solely those of the authors and do not necessarily represent those of their affiliated organizations, or those of the publisher, the editors and the reviewers. Any product that may be evaluated in this article, or claim that may be made by its manufacturer, is not guaranteed or endorsed by the publisher.
