# Supplementary material for: Effect of High Temperature Stress During the Reproductive Stage on Grain Yield and Nutritional Quality of Lentil (Lens culinaris Medikus)
Source: Front Nutr. 2022 Apr 15;9:857469. doi: 10.3389/fnut.2022.857469 (PMC9051399; doi:10.3389/fnut.2022.857469)
Supplement: Supplementary file 1 [file Table_1.docx]

**Table S1:** List of 36 tested genotypes their source and country of origin.

| IG | Origin | ID | Pedigree | DOI | Latitude | Longitude |
| --- | --- | --- | --- | --- | --- | --- |
| 224 | BEL | PI 238758 | NA | 10.18730/5N97A | NA | NA |
| 257 | IRN | PI 289069 | NA | 10.18730/5NA64 | 36.8 | 54.4 |
| 494 | GTM | PI 311107 | NA | 10.18730/5NHAA | 14.6 | -90.7 |
| 597 | RUS | PI 343025 | NA | 10.18730/5NM1Q | 54.3 | 48.3 |
| 619 | ARM | PI 345638 | NA | 10.18730/5NMG1 | 40.8 | 43.8 |
| 624 | MKD | PI 357226 | NA | 10.18730/5NMK4 | 41.4 | 21.9 |
| 918 | TUN | NEL 918 | NA | 10.18730/5NX0E | 36.8 | 10.2 |
| 950 | YEM | PI 244046 | NA | 10.18730/5NY09 | 13.6 | 44.0 |
| 956 | CHL | 33-032-10127 | NA | 10.18730/5NY6F | -36.6 | -72.1 |
| 1959 | ETH | EL 102 | NA | 10.18730/5PWT~ | 9.1 | 38.7 |
| 2181 | TUR | NA | ILL 182 selection | 10.18730/5Q1AX | 41.3 | 26.7 |
| 2230 | YUG | NA | ILL 624 selection | 10.18730/5Q2V4 | NA | NA |
| 3517 | IND | LG 46 | NA | 10.18730/5RA54 | 26.0 | 85.9 |
| 4345 | NA | 5091 | NA | 10.18730/5S2ZN | NA | NA |
| 4471 | SYR | SAMPLE NO. 27 | NA | 10.18730/5S6S* | 35.6 | 36.7 |
| 4605 | MAR | PRECOZ | NA | 10.18730/5SAWF | 35.8 | -5.5 |
| 4738 | CAN | ESTON | NA | 10.18730/5SF10 | NA | NA |
| 4791 | IRN | II-3-81 | NA | 10.18730/5SGPG | 38.4 | 47.1 |
| 4804 | LBY | II-3-135 | NA | 10.18730/5SH3X | 33.5 | 35.4 |
| 4841 | ALB | LENS 2 | NA | 10.18730/5SJ8X | 40.7 | 20.6 |
| 4881 | DEU | LENS 137 | NA | 10.18730/5SKG* | 52.7 | 12.3 |
| 5261 | JOR | UJL 42 | 80SH S19 UJL 32 | 10.18730/5SYGE | 32.4 | 35.9 |
| 5416 | ITA | NA | NA | 10.18730/5T3BN | 41.3 | 15.2 |
| 5505 | SDN | NA | NA | 10.18730/5T61~ | 19.2 | 30.5 |
| 5509 | SYR | 74TA 22 | ILL 31 selection | 10.18730/5T650 | 36.2 | 37.2 |
| 5562 | JOR | 76TA 66005 | ILL 1 selection | 10.18730/5T7TG | 32.1 | 36.1 |
| 5595 | SYR | 78S 26030 | ILL 25 selection | 10.18730/5T8VC | 33.5 | 36.3 |
| 69577 | CYP | ARI 240 | NA | 10.18730/7M0YS | 34.7 | 32.7 |
| 70056 | SYR-ICARDA | FLIP 86-15L | ILL 4349 x ILL 4605 | 10.18730/7MEMK | 54.6 | 39.7 |
| 70076 | SYR-ICARDA | FLIP 86-35L | ILL 4354 x ILL 922 | 10.18730/7MF82 | 54.6 | 39.7 |
| 71444 | MAR | LRT 55 | NA | 10.18730/7QD5A | 32.1 | -8.5 |
| 71479 | FRA | Mariette | NA | 10.18730/7QDAF | 35.2 | -5.3 |
| 71595 | SYR | OT-132-1 | NA | 10.18730/7QHTB | 37.0 | 41.5 |
| 73734 | SYR | LR 89 | NA | 10.18730/7SFMW | 35.8 | 36.7 |
| 73948 | ITA | VIR 1045 | NA | 10.18730/7SNV5 | 37.5 | 15.1 |
| LSI88 | NA | NA | NA | NA | NA | NA |
| IG, ICARDA Germplasm; ID, Identifier; DOI, Digital object identifier; NA, Not available.  BEL, Belgium; IRN, Iran; GTM, Guatemala; RUS, Russian Federation; ARM, Armenia; MKD, Macedonia; TUN, Tunisia; YEM, Yemen; CHL, Chile; ETH, Ethiopia; TUR, Turkey; YUG, Yugoslavia; IND, Indian; SYR, Syria; MAR, Morocco; CAN, Canada; LBY, Libya; ALB, Albania; DEU, Germany; JOR, Jordan; ITA, Italy; SDN, Sudan; CYP, Cyprus; FRA, France. | | | | | | |

**Table S2**: Combined analysis of variance for different traits among 36 lentil genotypes under no stress and heat stress condition

|  | df | FPP | UPP | TPP | GY | BY | HSW | SL | SW | SA | SP | SD | SE |
| --- | --- | --- | --- | --- | --- | --- | --- | --- | --- | --- | --- | --- | --- |
| Gen | 35 | 407.42** | 27.99* | 530.50** | 0.50** | 11.17** | 0.27** | 1.32** | 1.25** | 70.76** | 20.05** | 1.23** | 0.002** |
| Trt | 1 | 6094.62** | 1.94NS | 5879.11** | 16.85** | 581.90** | 3.65** | 1.05** | 1.16** | 75.42** | 20.80** | 1.42** | 0.01NS |
| GenxTrt | 34 | 238.88** | 46.96** | 329.36** | 0.42** | 4.06** | 0.31** | 0.11* | 0.11NS | 5.71NS | 1.85NS | 0.12NS | 0.002* |
| Rep | 1 | 0.001NS | 1.05NS | 1.13NS | 0.10NS | 0.39NS | 0.11NS | 0.04NS | 0.21NS | 5.77NS | 1.85NS | 0.15NS | 0.01NS |
| R^2^ | | 0.94 | 0.79 | 0.93 | 0.87 | 0.98 | 0.85 | 0.93 | 0.92 | 0.93 | 0.92 | 0.91 | 0.73 |
| FPP, Filled pods per plant; UPP, Unfilled pods per plant; TPP, Total pods per plant; GY, Grain yield; BY, Biological yield; HSW, hundred-seed weight; SL, Seed length; SW, Seed width; SA, Seed area; SP, Seed perimeter; SD, Seed diameter; SE, Seed eccentricity.  *, **, and NS indicate significance at 0.05 and 0.001 probability levels, and non-significant, respectively. | | | | | | | | | | | | |  |

**Table S2:** Continued

|  | df | FD | SC | ST | SR | CT | CP | PA | Zn | PA/Zn | Fe | PA/Fe |
| --- | --- | --- | --- | --- | --- | --- | --- | --- | --- | --- | --- | --- |
| Gen | 35 | 0.00** | 0.00* | 0.00** | 0.00** | 10.16** | 5.66** | 0.05** | 0.33* | 14.73** | 0.89** | 6.74** |
| Trt | 1 | 0.00NS | 0.00* | 0.00** | 0.002** | 166.51** | 89.25** | 0.28** | 16.93** | 343.04** | 59.60** | 228.79** |
| GenxTrt | 34 | 0.00* | 0.00* | 0.00* | 0.00NS | 8.64** | 5.01** | 0.07** | 0.61 ** | 19.28** | 1.19** | 9.81** |
| Rep | 1 | 0.01NS | 0.00NS | 0.00NS | 0.00NS | 6.96NS | 0.01NS | 1.97NS | 0.01NS | 6.01NS | 0.11NS | 2.28NS |
| R^2^ | | 0.71 | 0.68 | 0.76 | 0.87 | 0.92 | 0.91 | 0.87 | 0.82 | 0.84 | 0.92 | 0.9 |
| FD, Feret’s diameter; SC, Seed circularity; ST, Seed thickness; SR, Seed rugosity; CT, Cooking time; CP, Crude protein; PA, Phytic acid; Zn, Zinc content; PA/Zn, Phytic acid/Zinc ratio; Fe, Iron content; PA/Fe, Phytic acid/Iron ratio  *, **, and NS indicate significance at 0.05 and 0.001 probability levels, and non-significant, respectively. | | | | | | | | | | | | |

**Table S3:** Correlation coefficients between seed yield and seed shape parameters of 36 lentil genotypes under no stress (above diameter) and heat stress (below diameter) conditions.

|  | GY | HSW | SL | SW | SA | SP | SD | SE | SFD | SC | ST | SR |
| --- | --- | --- | --- | --- | --- | --- | --- | --- | --- | --- | --- | --- |
| GY | **1** | 0.628^**^ | 0.122 | 0.121 | 0.100 | 0.115 | 0.116 | 0.009 | 0.027 | -0.222 | 0.141 | -0.209 |
| HSW | 0.628^**^ | **1** | -0.077 | -0.089 | -0.109 | -0.083 | -0.083 | 0.072 | 0.143 | 0.010 | 0.034 | 0.033 |
| SL | 0.12 | -0.08 | **1** | 0.992^**^ | 0.989^**^ | 0.993^**^ | 0.992^**^ | -0.103 | -0.078 | -0.370^*^ | 0.751^**^ | -0.899^**^ |
| SW | 0.12 | -0.09 | 0.992^**^ | **1** | 0.992^**^ | 0.994^**^ | 0.994^**^ | -0.209 | -0.188 | -0.426^**^ | 0.725^**^ | -0.905^**^ |
| SA | 0.10 | -0.11 | 0.989^**^ | 0.992^**^ | **1** | 0.994^**^ | 0.993^**^ | -0.153 | -0.138 | -0.365^*^ | 0.719^**^ | -0.876^**^ |
| SP | 0.12 | -0.08 | 0.993^**^ | 0.994^**^ | 0.994^**^ | **1** | 1.000^**^ | -0.153 | -0.131 | -0.403^*^ | 0.754^**^ | -0.895^**^ |
| SD | 0.12 | -0.08 | 0.992^**^ | 0.994^**^ | 0.993^**^ | 1.000^**^ | **1** | -0.160 | -0.138 | -0.412^*^ | 0.757^**^ | -0.898^**^ |
| SE | 0.01 | 0.07 | -0.10 | -0.21 | -0.15 | -0.15 | -0.16 | **1** | 0.974^**^ | 0.547^**^ | 0.026 | 0.198 |
| SFD | 0.03 | 0.14 | -0.08 | -0.19 | -0.14 | -0.13 | -0.14 | 0.974^**^ | **1** | 0.557^**^ | 0.115 | 0.169 |
| SC | -0.22 | 0.01 | -0.370^*^ | -0.426^**^ | -0.365^*^ | -0.403^*^ | -0.412^*^ | 0.547^**^ | 0.557^**^ | **1** | -0.420^*^ | 0.570^**^ |
| ST | 0.14 | 0.03 | 0.751^**^ | 0.725^**^ | 0.719^**^ | 0.754^**^ | 0.757^**^ | 0.03 | 0.12 | -0.420^*^ | **1** | -0.797^**^ |
| SR | -0.21 | 0.03 | -0.899^**^ | -0.905^**^ | -0.876^**^ | -0.895^**^ | -0.898^**^ | 0.20 | 0.17 | 0.570^**^ | -0.797^**^ | **1** |
| GY, Grain yield (g); HSW, hundred-seed weight (g); SL, Seed length (mm); SW, Seed width (mm); SA, Seed area (mm^2^); SP, Seed perimeter (mm); SD, Seed diameter (mm); SE, Seed eccentricity (mm); SFD, Feret’s diameter; SC, Seed circularity; ST, Seed thickness; SR, Seed rugosity.  *Correlation is significant at the 0.05 level. **Correlation is significant at the 0.01 level. | | | | | | | | | | | | |
